# Monosaccharide transporter *OsMST6* is activated by transcription factor OsERF120 to enhance chilling tolerance in rice seedlings

**DOI:** 10.1093/jxb/erae123

**Published:** 2024-03-15

**Authors:** Shengtao Luo, Shuangshuang Zheng, Zhitao Li, Jie Cao, Bo Wang, Yunyuan Xu, Kang Chong

**Affiliations:** Key Laboratory of Plant Molecular Physiology, Institute of Botany, Chinese Academy of Sciences, Beijing 100093, China; University of Chinese Academy of Sciences, Beijing 100049, China; Key Laboratory of Plant Molecular Physiology, Institute of Botany, Chinese Academy of Sciences, Beijing 100093, China; University of Chinese Academy of Sciences, Beijing 100049, China; Key Laboratory of Plant Molecular Physiology, Institute of Botany, Chinese Academy of Sciences, Beijing 100093, China; University of Chinese Academy of Sciences, Beijing 100049, China; University of Chinese Academy of Sciences, Beijing 100049, China; Key Laboratory of Plant Molecular Physiology, Institute of Botany, Chinese Academy of Sciences, Beijing 100093, China; University of Chinese Academy of Sciences, Beijing 100049, China; Key Laboratory of Plant Molecular Physiology, Institute of Botany, Chinese Academy of Sciences, Beijing 100093, China; University of Chinese Academy of Sciences, Beijing 100049, China; Key Laboratory of Plant Molecular Physiology, Institute of Botany, Chinese Academy of Sciences, Beijing 100093, China; University of Chinese Academy of Sciences, Beijing 100049, China; University of California, Davis, USA

**Keywords:** Chilling tolerance, glucose, monosaccharide transporter, OsMST6, OsERF120, rice

## Abstract

Chilling stress caused by extreme weather is threatening global rice (*Oryza sativa* L.) production. Identifying components of the signal transduction pathways underlying chilling tolerance in rice would advance molecular breeding. Here, we report that *OsMST6*, which encodes a monosaccharide transporter, positively regulates the chilling tolerance of rice seedlings. *mst6* mutants showed hypersensitivity to chilling, while *OsMST6* overexpression lines were tolerant. During chilling stress, OsMST6 transported more glucose into cells to modulate sugar and abscisic acid signaling pathways. We showed that the transcription factor OsERF120 could bind to the DRE/CRT element of the *OsMST6* promoter and activate the expression of *OsMST6* to positively regulate chilling tolerance. Genetically, OsERF120 was functionally dependent on OsMST6 when promoting chilling tolerance. In summary, OsERF120 and OsMST6 form a new downstream chilling regulatory pathway in rice in response to chilling stress, providing valuable findings for molecular breeding aimed at achieving global food security.

## Teaser

In a novel downstream chilling regulatory pathway, the binding of *OsMST6* promoter, by a new ethylene-responsive transcription factor, OsERF120, enhances chilling tolerance in rice seedlings.

## Introduction

Rice (*Oryza sativa* L.) is one of the most important staple crops and feeds nearly half of the world's population (Li *et al.*, 2023). It originates from tropical and subtropical regions and is sensitive to low temperatures, and as such, chilling stress limits the sowing time and geographical distribution of rice (Zhang *et al.*, 2017). In high-latitude or high-altitude regions, rice can be severely impacted by cold during the seedling stage, leading to delayed development or even seedling death, ultimately affecting rice yield (Zhang *et al.*, 2014; Zhang *et al*., 2019). Exploring the chilling tolerance network is a powerful base for molecular breeding aimed at promoting increased and stable rice yields.

In rice cells, sensors detect chilling stress, triggering the cold signaling network to activate tolerance mechanisms. For example, the interaction between COLD1 and RGA1 triggers calcium influx, which activates multiple transcription factors, such as the dehydration responsive element binding (DREB) transcription factors (Ma *et al.*, 2015; Guo *et al.*, 2018). DREB belongs to the APETALA2 (AP2)/ethylene-responsive element binding factor (ERF) superfamily, which is divided into four subfamilies: AP2, RAV, DREB, and ERF (Feng *et al.*, 2020). In Arabidopsis, *CBF1/DREB1B*, *CBF2/DREB1C*, and *CBF3/DREB1A* are induced by chilling stress. The expression of *DREB1A* and *DREB1B* is transiently and tightly controlled by DREB1C (Novillo *et al.*, 2004). In rice, *OsDREB1A* is also induced by chilling stress, and its overexpression results in enhanced tolerance to freezing, drought, and salt stresses (Dubouzet *et al.*, 2003). ERFs, distinguished from DREBs by the 14th and 19th amino acids, have been reported to be primarily associated with drought stress. AtTINY, a member of the ERF transcription factor subfamily, has been shown to positively regulate drought responses in Arabidopsis (Xie *et al.*, 2019). The rice ERF transcription factor OsERF101 has also been shown to positively regulate drought tolerance during the vegetative and reproductive stages (Jin *et al.*, 2018). Another rice ERF transcription factor, OsERF71, directly binds to the promoter of *OsCCR1*, an essential gene involved in lignin biosynthesis, resulting in morphological modifications of roots that enhance drought resistance (Lee *et al.*, 2016). Although numerous studies have detailed the relationship between DREBs and chilling tolerance, minimal research has been done to elucidate the functional roles of ERFs in chilling stress responses in rice.

Osmotic regulation plays a vital role in plant responses to chilling stress. Chilling stress leads to the accumulation of solutes such as proline, soluble sugars (sucrose, glucose, trehalose, and fructose), and polyols (mannitol and sorbitol) that protect plant cells from chilling damage (Zulfiqar *et al.*, 2019). For example, the expression of *OsTPP1*, involved in the trehalose synthesis pathway, is up-regulated by OsHLH002/OsICE1 in response to chilling stress, leading to increased trehalose accumulation and resistance to chilling damage (Zhang *et al.*, 2017). Glucose not only serves as a potent osmoprotectant during chilling stress, but also functions as a precursor for multiple metabolic processes and signaling molecules (Saddhe *et al.*, 2021). Detection of glucose by hexokinases (HXKs) activates downstream signal pathways and HXK catalyses its conversion to glucose 6-phosphate for the synthesis of polyols (Sakr *et al.*, 2018). Additionally, glucose can interact with various plant hormones to coordinately regulate plant responses to abiotic stresses (Lee and Seo, 2015). Despite numerous studies on the role of sugars, there is still a lack of knowledge regarding the mechanisms and pathways through which sugars are transported into cells during chilling stress.

Sugar transport in plants requires the participation of multiple transporters, including the monosaccharide transporter (MST) (Kong *et al.*, 2019). The MST family comprises seven subfamilies, known as ERD6, pGlcT, INT, VGT, TST, PLT, and STP (Saddhe *et al.*, 2021). Sugar transporter protein (STP) is regarded as an H^+^–sugar symporter, and harbors 12 transmembrane domains (Deng *et al.*, 2019). The STP subfamily contains 14 members in Arabidopsis and 28 members in rice. Several STPs have been found to be involved in abiotic and biotic stress responses (Kong *et al.*, 2019). In Arabidopsis, *AtSTP13* is induced by drought and salt stress (Yamada *et al.*, 2011). Phosphorylation of AtSTP13 by Bri1-associated kinase receptor 1 (BAK1) at Thr485 enhances its monosaccharide uptake activity, resulting in enhanced antibacterial defense responses (Yamada *et al.*, 2016). In rice, the expression of *OsMST8/OsSTP8* is significantly repressed by chilling stress in anthers (Mamun *et al.*, 2006). Seven *OsSTP* members, *OsMST2/OsSTP2*, *OsMST3/OsSTP3*, *OsMST4/OsSTP4*, *OsSTP11*, *OsSTP19*, *OsSTP25,* and *OsSTP28*, are significantly up-regulated in rice seedlings exposed to salt, osmotic, and drought stresses (Deng *et al.*, 2019). OsMST6, also named OsSTP6, is a broad-spectrum monosaccharide transporter and can be induced by salt, glucose, and sucrose treatments (Wang *et al.*, 2008). Heterologous overexpression of *OsMST6* enhances drought and salt tolerance in Arabidopsis (Monfared *et al.*, 2020). However, it remains unknown how *OsMST6* affects chilling tolerance in rice.

In this study, we identified a monosaccharide transporter gene, *OsMST6*, that played a critical role in chilling stress responses in rice seedlings. We demonstrated that *mst6* mutants exhibited increased cold sensitivity, whereas *OsMST6* overexpression lines exhibited enhanced cold tolerance. We detailed the molecular function of this response, wherein activation of *OsMST6* by OsERF120 stimulated glucose transport to enhance rice seedling chilling tolerance.

## Materials and methods

### Plant materials

The mutant and overexpression lines in this study were derived from *Oryza sativa* ssp. *japonica* cv. Zhonghua11 (ZH11). *mst6-1* and *mst6-2* mutants were generated through the CRISPR/CAS9 genome editing system, according to our previous study (Li *et al.*, 2023). The guide sequence of *OsMST6* was designed using the E-CRISP website (http://www.e-crisp.org/). Oligonucleotides containing the guide sequences were synthesized and annealed to form double-stranded DNA. The annealed oligonucleotides were then cloned into the pCRISPR vector, which had been linearized by *Bsa*I. The *erf120-1* and *erf120-2* mutants were generated through the dual-targeted CRISPR/CAS9 genome editing system (Biogle, cat. no. BGK03) according to the manufacturer’s instructions. The *OsERF120* guide sequences were designed using the E-CRISP website. After PCR amplification using pU3 as the template and the synthesized guide sequences as primers, the products were cloned into the pCRISPR-II vector.

To generate overexpression lines, full-length cDNAs of *OsMST6* and *OsERF120*, which were amplified from the ZH11 cDNA, were cloned into the pCAMBIA1300 vector under the control of the constitutive cassava vein mosaic virus (CsVMV) promoter. The resulting plasmid constructs were introduced into ZH11 by *Agrobacterium tumefaciens*-mediated transformation. The primers used in this study are listed in Supplementary Table S1.

### Rice growth conditions and chilling treatment

The rice growth conditions and chilling treatments at the seedling stage in this study follow those described in our previous studies (Zhang *et al.*, 2017; Li *et al.*, 2021, 2023). Rice seeds were soaked in water at 30 °C for 3 d, then germinated rice seeds were transferred into 96-well plates. Seedlings were grown in Kimura B nutrient solution in a greenhouse for 2 weeks. The optimization of environmental control parameters in the greenhouse was set as follows: photoperiod (10 h: 14 h, light: dark) and temperature (28 °C: 25 °C, light: dark). Seedlings at the three-leaf stage were subjected to the chilling treatment by incubating them in a water bath, maintained precisely at 4 ± 0.3 °C. The *mst6* mutants were chilling-treated for 84 h, the *erf120* mutants for 90 h, and the overexpression lines for 108 h. After the chilling treatment, seedlings were transferred back to the greenhouse to recover for 2 weeks and the survival rates were recorded. Each experiment was performed at least three times independently.

Chilling treatment of rice at the booting stage was conducted according to the following procedure (Liu *et al.*, 2024). Seeds were soaked in water at 30 °C for 3 d, then germinated seeds were planted in nutrient-rich soil for seedling cultivation. Seedlings were transplanted into mobile containers containing a mixture of soil and vermiculite, and cultivated under normal field management conditions. Once the rice plants reached the meiosis stage of pollen development, panicles were labeled, and the containers were transferred to water kept at 17–18 °C, maintained at a depth of 30–40 cm above the soil surface. After about 2 weeks, plants that had completed pollination and fertilization were transferred back to the original field. The seed-setting rate of rice was used as the metric to evaluate chilling tolerance. Each experiment was performed at least three times independently.

### Evaluation of agronomic traits

The seeds of *OsMST6* mutants, overexpression lines, and the wild-type (WT) ZH11 were harvested from the field simultaneously and stored under identical conditions. The thousand-grain weight was determined by weighing 1000 randomly selected dried seeds. The seed setting rate was determined by calculating the ratio of filled seeds to the total number of seeds of randomly selected rice plants. The grains per panicle was determined by counting the total number of grains on randomly selected panicles. The experiments were repeated at least three times independently. The values represent means ±SD of three biological replicates.

### Subcellular localization of OsMST6 and OsERF120

Subcellular localization assays were carried out using a transient expression system in rice protoplasts, according to previously reported methods (Chen *et al.*, 2006). Full length cDNAs of *OsMST6* and *OsERF120* were cloned into the pBI221 vector with a green fluorescent protein (GFP) tag at the C-terminus to construct the *35S::OsMST6-GFP* and *35S::OsERF120-GFP* constructs. The *35S::OsMST6-GFP* was co-transformed with a plastid membrane marker, *35S::PIP2-RFP*, into rice protoplasts derived from 10-day-old etiolated seedling sheaths using the polyethylene glycol (PEG)-mediated transformation method. As a control, the pBI221 empty-vector was co-transformed with *35S::PIP2-RFP*. The *35S::OsERF120-GFP* was co-transformed with *35S::PIP2-RFP* or a nuclear marker, *35S::H2B-RFP*. The transformed protoplasts were visualized using a fluorescence microscope (Olympus FV1000MPE).

### Total RNA extraction and quantitative RT-PCR

Total RNA was extracted from whole seedlings using the KK Fast Plant Total RNA Kit (Zoman, cat. no. ZP405K). First-strand cDNA synthesis was performed with 2 μg of total RNA, using the Hifair^®^ II 1st strand cDNA Synthesis Supermix for qPCR (Yeasen, cat. no. 11123ES60), and the resulting cDNA was diluted at a 1:30 ratio. Quantitative PCR reactions were run on the Applied Biosystems QuantStudio 3 Real-Time PCR System (Thermo Fisher Scientific), using the Hieff qPCR SYBR Green Master Mix (Yeasen, cat. no. 11202ES08). Gene expression levels were analysed using the $2_{}^{-\Delta \Delta C_{T}}$ method and normalized by the *Ubiquitin* and *Actin* genes. Each experiment included three technical replicates and three biological replicates. The values represent means ±SD of three biological replicates. The primers used are listed in Supplementary Table S1.

### Flow cytometry assays

Flow cytometry assays were performed as previously with minor modifications (Zou *et al.*, 2005). Protoplasts were obtained from the leaf sheaths of 10-day-old ZH11, *mst6-1*, and OE1 etiolated seedlings. Rice protoplasts were incubated at 4 °C in the 100 μmol l^–1^ solution of 2-[*N*-(7-nitrobenz-2-oxa-1,3-diazol-4-yl) amino]-2-deoxy-d-glucose (2-NBDG), which is a fluorescent d-glucose analog. After 1 h incubation, protoplasts were washed three times with pre-chilled W5 buffer (154 mmol l^–1^ NaCl, 125 mmol l^–1^ CaCl_2_, 5 mmol l^–1^ KCl, 2 mmol l^–1^ MES pH 5.7) to remove excess 2-NBDG. Protoplasts were resuspended in 200 μl of W5 buffer and kept on ice. For each measurement, data from about 10 000 cell events were collected using the Moflo XDP FACS sorter (Beckman Coulter). The fluorescence values represent means ±SD of three biological replicates.

### Sugar quantification assays

Apoplast sugar extraction assays were performed as previously reported, with minor modifications (Yamada *et al.*, 2016). After the 14-day cultivation, rice seedlings at the three-leaf stage were washed thoroughly three times with deionized water. Subsequently, roots were cut, blotted dry, and placed into deionized water. The samples were submerged in water, and then a vacuum was applied for 15 min, after which the samples were removed, blotted dry, then weighed and recorded as M1. The samples were wrapped with Parafilm and placed in a 10 ml syringe, which was placed in centrifuge tube. Centrifugation was done at 5000 *g* for 10 min to collect the intercellular fluid at the bottom of the centrifuge tubes. Intercellular fluid was collected for glucose 6-phosphate dehydrogenase (G6PDH) activity measurement to quantify the degree of cytoplasmic contaminants (Comin, cat. no. G6PDH-1-Y) and apoplast sugar measurement. The remaining samples were weighed again and recorded as M2. The obtained mass of the intercellular fluids was represented by M2−M1. If the degree of G6PDH in the intercellular wash fluid were similar to that of the water control, apoplast sugar levels were measured using a sucrose/d-fructose/d-glucose test kit (Megazyme, cat. no. K-SUFRG). The values represent means ±SD of three biological replicates.

### Yeast one-hybrid assays

Yeast one-hybrid assays were used to examine the binding of transcription factors to the *OsMST6* promoter, according to our previously described method (Zhang *et al.*, 2017). Full length cDNAs of the transcription factors (*OsERF120*, *OsERF121*, *OsERF013*, *OsDREB1A*, *OsDREB1B*, and *OsDREB1D*) were cloned into the pGAD42 vector, and the 2 kb promoter sequence upstream of *OsMST6* was cloned into the pLacZi vector. These fused plasmids were co-transformed into *Saccharomyces cerevisiae* strain EGY48. After culturing at 30 °C for 3 d, the yeast transformants were selected and incubated overnight in SD medium (−Trp−Ura). The OD_600_ was adjusted to 1.0 before dotting 3 μl of serial diluted (1×, 10×, and 100×) cultures onto SC plates (−Trp−Ura) containing 5-bromo-4-chloro-3-indolyl-β-d-galactopyranoside (X-Gal), with galactose and raffinose as carbon sources. The yeast transformants were again cultured at 30 °C for 3 d, and the blue color development was observed. The experiment was performed at least three times independently.

### Electrophoretic mobility shift assay assays

The OsERF120 protein purification assays were performed according to our previously described methods (Guo *et al.*, 2023). *OsERF120* was cloned into the pMAL-C4X vector, then transformed into the *Escherichia coli* strain BL21 (DE3) cells, and induced with 0.5 mmol l^–1^ isopropyl β-d-thiogalactoside induced for 16 h at 16 °C. Cells were collected by centrifugation at 5000 *g* for 10 min at 4°C. The cells were lysed by sonication in lysis buffer (50 mmol l^–1^ Tris–HCl, 100 mmol l^–1^ NaCl, 1 mmol l^–1^ EDTA, pH 7.5). The MBP–OsERF120 fused protein was purified using Amylose Resin (New England Biolabs, cat. no. E8021L) and eluted with a 20 mmol l^–1^ maltose in 1× phosphate-buffered saline solution.

The 29 bp oligonucleotide sequence flanking the dehydration response element/C-repeat (DRE/CRT) element, from position −533 to −505 in the *OsMST6* promoter, was synthesized and utilized as the probe. Oligonucleotides with one, two, and three base mutations in the DRE/CRT element were separately synthesized, resulting in mutation probe 1, mutation probe 2, and mutation probe 3. All probes were labeled with biotin at the 5ʹ end. The competitor probe possessed the same sequence as the probe, but lacked biotin labeling. To anneal to dsDNA, the oligonucleotides were incubated in annealing buffer (100 mmol l^–1^ NaCl, 1 mmol l^–1^ EDTA, 10 mmol l^–1^ Tris–HCl pH 7.5) at 65 °C for 10 min, then slowly cooled down. The protein–DNA reaction and chemiluminescent nucleic acid detection were conducted using the LightShift Chemiluminescent EMSA Kit (Thermo Fisher Scientific, cat. no. 20148). The experiment was performed at least three times independently.

### Transcriptional activity assays

Transcription activity assays were carried out using a transient expression system in rice protoplasts, according to previously reported methods (Li *et al.*, 2021). The *35S::OsERF120-GFP* construct was used as the effector of transcription activity assays. The *OsMST6* promoter and the *OsMST6Δ* promoter, lacking the ‘CCGAC’ motif, were fused to the luciferase (LUC) reporter gene to generate *OsMST6::LUC* and *OsMST6Δ::LUC* reporter constructs. The reporter constructs also included *35S::GUS* as the normalization control. The *35S::OsERF120-GFP* construct was separately co-transformed with the *OsMST6::LUC* and *OsMST6Δ::LUC* constructs into rice protoplasts obtained from 10-day-old etiolated seedling sheaths, using the PEG method. After incubating at room temperature for 16 h, the protoplasts were centrifuged at 300 *g* for 3 min, then the supernatant was removed, and lysis buffer was added to the pellet and vigorously shaken. The samples were centrifuged at 1000 *g* for 2 min at 4 °C, and the supernatant was collected for the subsequent measurements. The luciferase activity was measured using the Luciferase assay system (Promega, cat. no. E1500) on the GloMax 20/20 Luminometer (Promega), while β-glucuronidase (GUS) activity was measured by mixing with MUG buffer (1 mmol l^–1^ 4-methylumbelliferyl-β-d-glucuronide, 0.1% (v/v) Triton X-100, 0.1% (w/v) sarkosyl, 10 mmol l^–1^ EDTA, 10 mmol l^–1^ β-mercaptoethanol, 50 mmol l^–1^ sodium phosphate buffer pH 7.0). The LUC/GUS ratios were used to determine transcriptional activity. The values represent means ±SD of three biological replicates.

### RNA-seq analysis

Rice seedlings of the WT ZH11 and the *mst6-1* mutant were cultivated for 2 weeks in Kimura B nutrient solution in the greenhouse. The 14-day-old rice seedlings were chill-treated for 0 h and 4 h at 4 °C, then the entire seedlings were sampled for transcriptome sequencing with three biological replicates. RNA-seq was performed at Anoroad Genome (Beijing, China). Clean reads were mapped referring to the rice genome (*Oryza sativa* IRGSP-1.0.38), using HISAT2 (Sirén *et al.*, 2014) with default parameters. Significant differentially expressed genes were determined using DESeq2 with |fold change| ≥2 and adjusted *P*-value <0.05. Gene ontology (GO) analysis was performed using DAVID (Huang *et al.*, 2009).

### Abscisic acid treatment

To assess the impact of abscisic acid (ABA) treatment on the chilling tolerance of rice seedlings, ZH11, *mst6-1*, and *mst6-2* rice seedlings at the three-leaf stage were sprayed with a 100 μmol l^–1^ ABA solution, while the control group was sprayed with deionized water (Liu *et al.*, 2018). After 24 h, the seedlings were transferred to a 4 °C water bath for 72 h, then returned to the greenhouse to recover for 14 d. The growth status of each plant seedling was assessed and scored based on the Standard Evaluation System (SES) by IRRI (1, seedlings dark green; 3, seedlings light green; 5, seedlings yellow; 7, seedlings brown; 9, seedlings dead) (Kim and Tai, 2011). The values represent means ±SD of 25 individual plants.

### ABA quantification

Fourteen-day-old rice seedlings of the ZH11, *mst6-1*, and OE1 lines that were cultivated in a greenhouse as described above, were incubated for 0 h and 4 h at 4 °C, then the entire seedlings were sampled for ABA quantification with three biological replicates. Each sample, consisting of five to eight seedlings with a mass exceeding 500 mg, was precisely weighed after being ground in liquid nitrogen. Subsequently, 10 ml of acetonitrile and 8 ng of ABA internal standard (Sigma-Aldrich) were added. The extraction process was carried out overnight at 4 °C. The samples were centrifuged at 12 000 *g* for 5 min at 4 °C, and the supernatants were collected. The extraction process was repeated once, and then the supernatants were combined and dried in nitrogen gas, before being redissolved in methanol for subsequent quantification of ABA content via HPLC-MS/MS analysis at Ruiyuan Biotechnology (Nanjing, China).

## Results

### *OsMST6* responds to chilling tolerance in rice

**Fig. 1.**
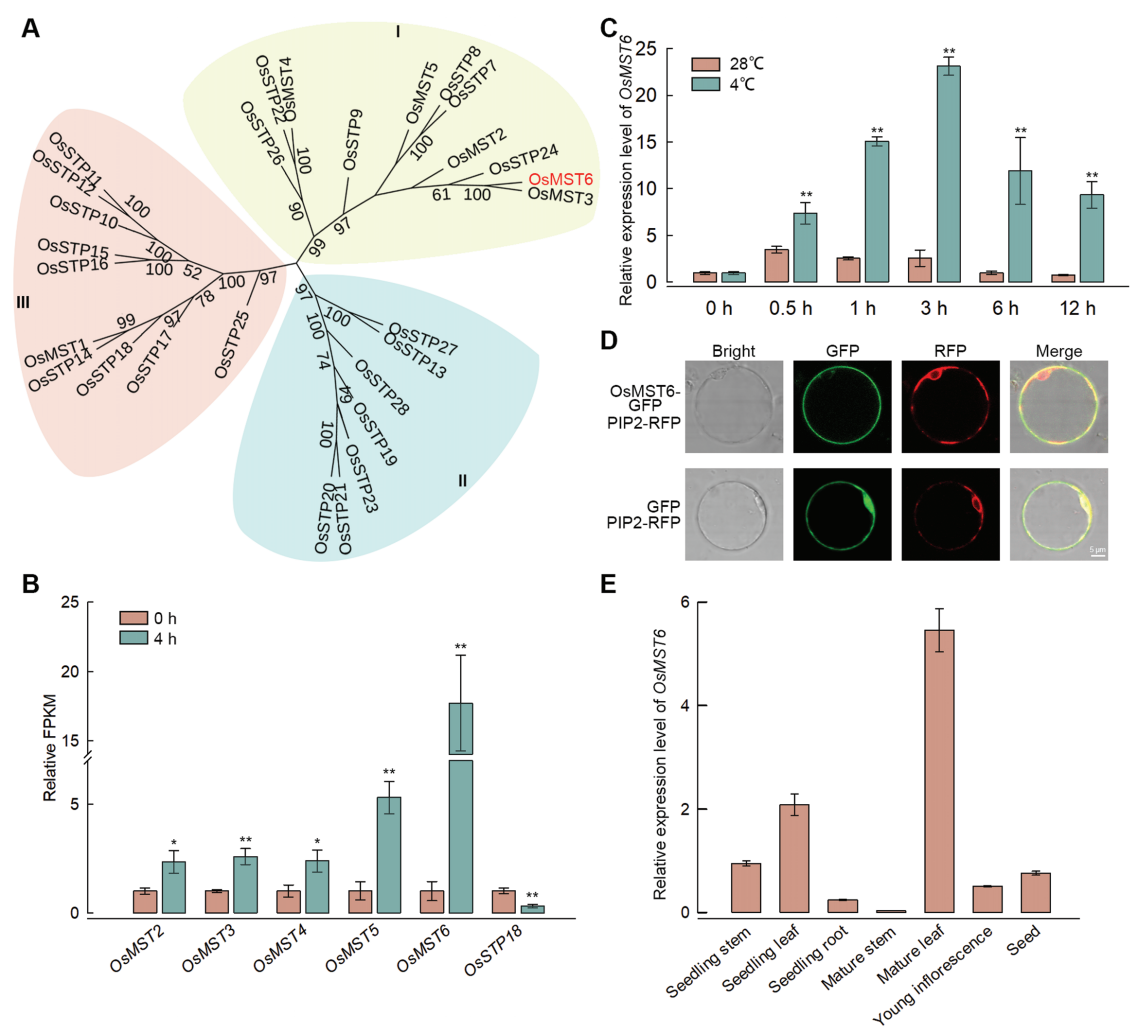
Phylogenetic and gene expression analysis of *OsMST6*. (A) Phylogenetic tree of the STP subfamily in rice. (B) Relative fragments per kilobase per million mapped reads (FPKM) levels after 0 h and 4 h of chilling treatment in the ZH11 transcriptome. Data are means ±SD (*n=*3). (C) Relative expression levels after chilling treatment for 0, 0.5, 1, 3, 6, and 12 h. The expression level of *OsMST6* at 0 h was set as ‘1’. Data are means ±SD (*n=*3). (D) Subcellular localization of OsMST6. PIP2–RFP was used as a plastid membrane marker. Scale bar, 5 μm; GFP, green fluorescent protein; RFP, red fluorescent protein. (E) qRT-PCR analysis of expression pattern of *OsMST6* in different tissues. The expression level of *OsMST6* in the seedling stem was set as ‘1’. Data are means ±SD (*n=*3). Significant differences were determined by Student’s *t*-test: *P<0.05, **P<0.01.

Based on phylogenetic analysis of gene sequences, the STP subfamily, which consisted of 28 members, can be divided into three clusters (Fig. 1A). The transcriptomic analysis of rice seedlings showed that only *OsMST2*, *3*, *4*, *5*, and *6*, which were grouped in cluster I, were up-regulated after the 4 h chilling treatment (Fig. 1B), with *OsMST6* exhibiting the most significant increase in expression. qRT-PCR results showed that the expression levels of *OsMST6* in rice seedlings incubated at 4 °C increased by 2.1-, 5.9-, 9.0-, 12.1-, and 12.3-fold at 0.5, 1, 3, 6, and 12 h, respectively, relative to that in seedlings grown at 28 °C (Fig. 1C). The expression of *OsMST6* first increased and then decreased, reaching peak levels at 3 h of chilling stress. To determine the subcellular localization of OsMST6, the OsMST6–GFP fusion protein was transiently co-expressed with PIP2–RFP in rice protoplasts. The results showed that OsMST6 mainly co-localized with PIP2 in the plastid membrane (Fig. 1D). Tissue-specific expression patterns showed that OsMST6 was preferentially expressed in the seedling leaf and mature leaf, while the expression was lowest in the mature stem (Fig. 1E).

**Fig. 2.**
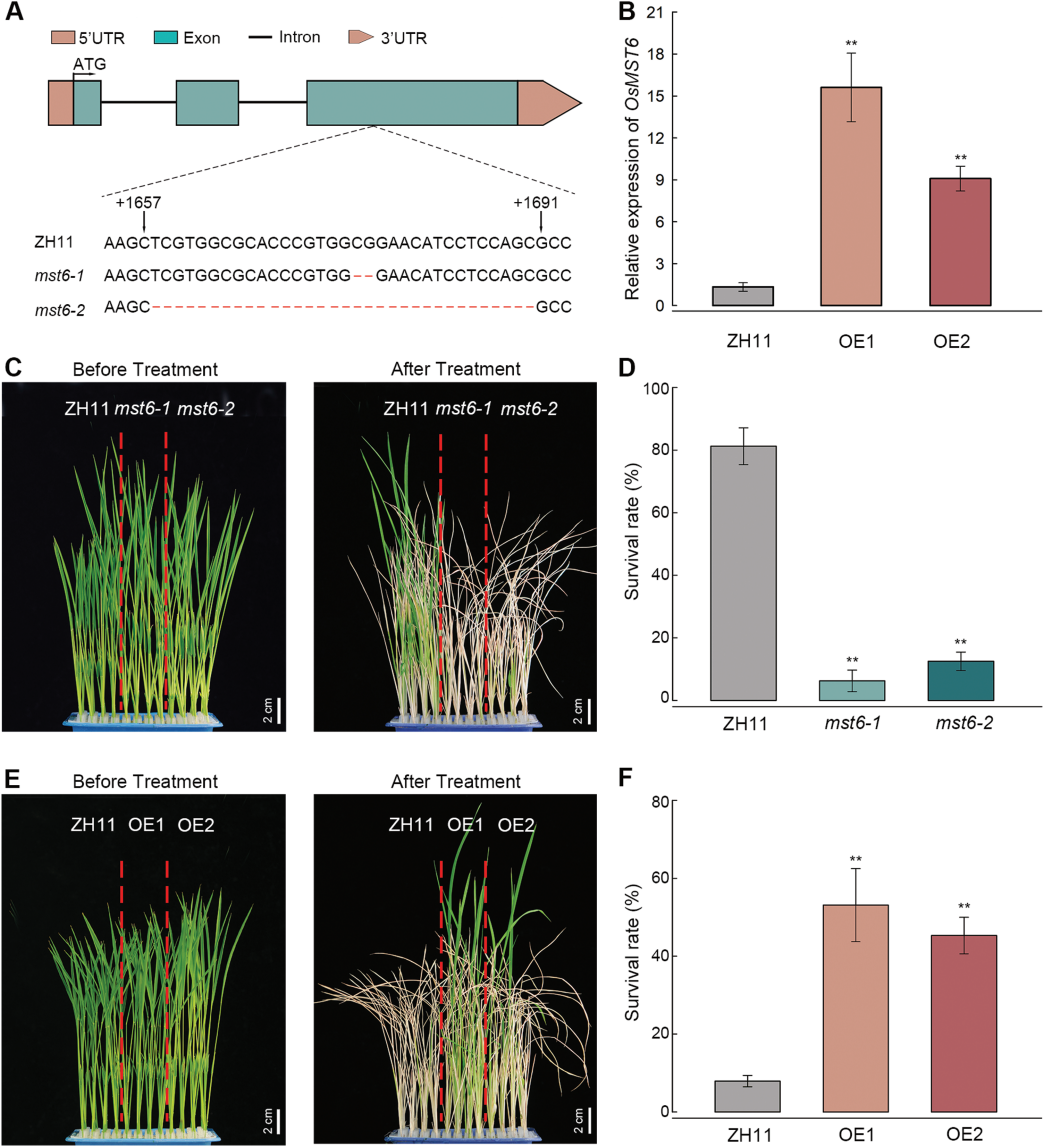
Chilling tolerance of *OsMST6* transgenic seedlings. (A) The gene structure and mutation sites of *mst6-1* and *mst6-2*. (B) Relative expression levels of *OsMST6* in the *OsMST6* overexpression lines (OE1, OE2) and the WT ZH11. The expression level of *OsMST6* in the ZH11 was set as ‘1’. Data are means ±SD (*n*=3). (C) Phenotypes of ZH11, *mst6-1*, and *mst6-2* lines after chilling treatment. Scale bars, 2 cm. Fourteen-day-old seedlings were incubated at 4 °C for 84 h and then returned to the greenhouse for recovery. (D) The survival rate of seedlings after 84 h of chilling treatment. Data are means ±SD (*n*>25 for each replicate). (E) Phenotypes of the ZH11, OE1, and OE2 lines after chilling treatment. Scale bars, 2 cm. Fourteen-day-old seedlings were incubated at 4 °C for 108 h and then transferred to the greenhouse for recovery. (F) The survival rate of seedlings after 108 h of chilling treatment. Data are means ±SD (*n>*25 for each replicate). Significant differences were determined by Student’s *t*-test, **P<0.01.

To understand the genetic function of *OsMST6*, two knockout mutant lines (*mst6-1* and *mst6-2*) and two overexpression lines (OE1 and OE2) were generated in the Zhonghua 11 (ZH11) background. DNA sequencing results showed that *mst6-1* and *mst6-2* had 2- and 33-nucleotide deletions in the third exon, respectively (Fig. 2A). As a result, there was a frame-shift mutation in *mst6-1* and the loss of 11 amino acids in *mst6-2* (Supplementary Fig. S1). qRT-PCR results showed that the transcript levels of *OsMST6* increased 15.6- and 9.1-fold in OE1 and OE2 lines, respectively, compared with the ZH11 (Fig. 2B). To evaluate the effects of *OsMST6* on chilling tolerance, seedlings of the *mst6-1*, *mst6-2*, and WT lines were incubated at 4 °C for 84 h, then returned to the greenhouse for recovery. The survival rate of the ZH11 seedlings was 81%, while those of the *mst6-1* and *mst6-2* mutants were 13% and 6%, respectively (Fig. 2C, D). When the seedlings were incubated at 4 °C for 108 h, the survival rates of OE1 (53%) and OE2 (45%) were higher than that of ZH11 (8%) (Fig. 2E, F). These results suggest that *OsMST6* responds to chilling stress and positively regulates chilling tolerance in rice seedlings.

Chilling tolerance evaluation assays were also conducted with rice plants at the booting stage, and we found that the *mst6-1* and *mst6-2* mutants were sensitive to chilling stress (Supplementary Fig. S2A). Statistical analysis of agronomic traits of the ZH11, *mst6-1*, *mst6-2*, OE1, and OE2 lines showed no significant differences between the overexpression lines and ZH11. However, the mutants exhibited an increase in 1000-grain weight, compared with ZH11 (Supplementary Fig. S2B); meanwhile, the seed-setting rate and grains per panicle significantly decreased (Supplementary Fig. S2C, D). These findings imply that *OsMST6* plays a positive regulatory role in the chilling tolerance during the rice booting stage and has an impact on grain yields.

### OsMST6 affects glucose transportation in response to chilling stress

**Fig. 3.**
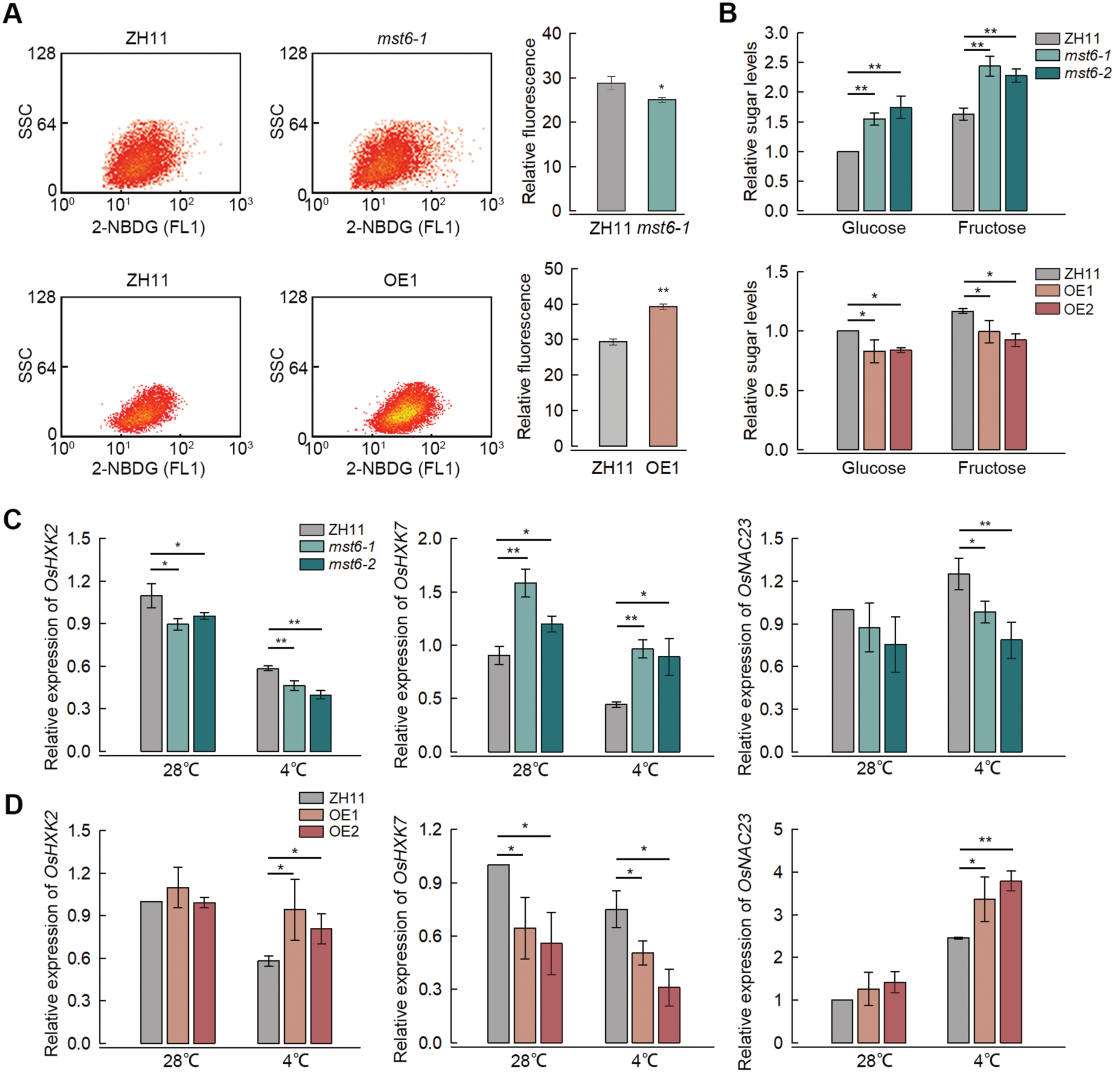
OsMST6 affects glucose transport in response to chilling stress. (A) Relative fluorescence intensity of ZH11, *mst6-1*, and OE1 protoplasts after incubation in 100 μmol l^–1^ of 2-[*N*-(7-nitrobenz-2-oxa-1,3-diazol-4-yl) amino]-2-deoxy-d-glucose (2-NBDG) for 1 h at 4 °C. About 10 000 cells were counted and analysed by flow cytometry. 2D plots indicate the relative granularity or internal complexity (side scatter, SSC) and relative 2-NBDG fluorescence intensity, detected in the FL1 channel, of protoplasts. Data are means ±SD (*n*=3). (B) Relative apoplastic glucose and fructose content in ZH11, *mst6*, and OE lines after chilling treatment for 24 h. Data are means ±SD (*n*=3). (C, D) Relative expression of sugar signaling-related genes *OsHXK2*, *OsHXK7*, and *OsNAC23* in *OsMST6* mutants (C) and overexpression lines (D) after chilling treatment for 12 h. The expression level of genes in ZH11 at 28 °C was set as ‘1’. Data are means ±SD (*n*=3). Significant differences were determined by Student’s *t*-test, *P<0.05, **P<0.01.

To investigate the role of OsMST6 in glucose transportation in rice, the d-glucose fluorescent analog 2-NBDG was used as a reporter substrate. Rice protoplasts were incubated in a 100 μmol l^–1^ solution of 2-NBDG for 1 h at 4 °C, and the fluorescence intensities from 10 000 cells were detected by flow cytometry. Under the 4 °C chilling treatment, the fluorescence intensity in ZH11 protoplasts reached 28.7, whereas it was significantly reduced in *mst6-1* protoplasts (25.0). Additionally, while the fluorescence intensity in ZH11 protoplasts reached 30.2, OE1 exhibited a higher fluorescence intensity of 38.6 (Fig. 3A; Supplementary Fig. S3). These data imply that OsMST6 can transport glucose into protoplasts.

When rice seedlings were subjected to chilling treatment for 24 h, the glucose content of entire seedlings was measured, and no significant difference was observed between the ZH11 and the *mst6* mutants. Subsequently, the levels of glucose and fructose in apoplasts of the *mst6*, OE, and ZH11 lines were measured after 24 h chilling treatment. The results revealed that after a 24 h chilling treatment, the *mst6-1* and *mst6-2* mutant lines exhibited higher concentrations of glucose and fructose in the apoplasts compared with ZH11. Conversely, glucose and fructose concentrations in the apoplasts of the OE1 and OE2 lines were significantly lower than those in ZH11 (Fig. 3B). These results suggest that OsMST6 affects glucose transport in rice seedlings.

To investigate whether glucose transport into cells affected sugar signaling, the expression levels of *OsHXK2*, *OsHXK7*, and *OsNAC23* were examined after chilling treatment. *OsHXK2* and *OsHXK7* belong to the hexokinase family, which are sugar sensors in rice, and are up- and down-regulated, respectively, by glucose treatment (Cho *et al.*, 2006; Kim *et al.*, 2016). qRT-PCR results showed that the expression levels of *OsHXK2* were lower in the *mst6-1* and *mst6-2* lines compared with ZH11 after chilling treatment, while they were higher in the OE1 and OE2 lines. In contrast, the expression pattern of *OsHXK7* showed the opposite trend (Fig. 3C, D). *OsNAC23* was reported to regulate sugar homeostasis, and its expression is positively correlated with sugar levels in rice (Li *et al.*, 2022). The qRT-PCR results demonstrated that *OsNAC23* was down-regulated in the mutants after chilling treatment, while it was up-regulated in the overexpression lines (Fig. 3C, D). These results imply that *OsMST6* affects the sugar signaling pathway in rice seedlings exposed to chilling stress.

### OsERF120 binds the promoter of *OsMST6* and activates its transcription

**Fig. 4.**
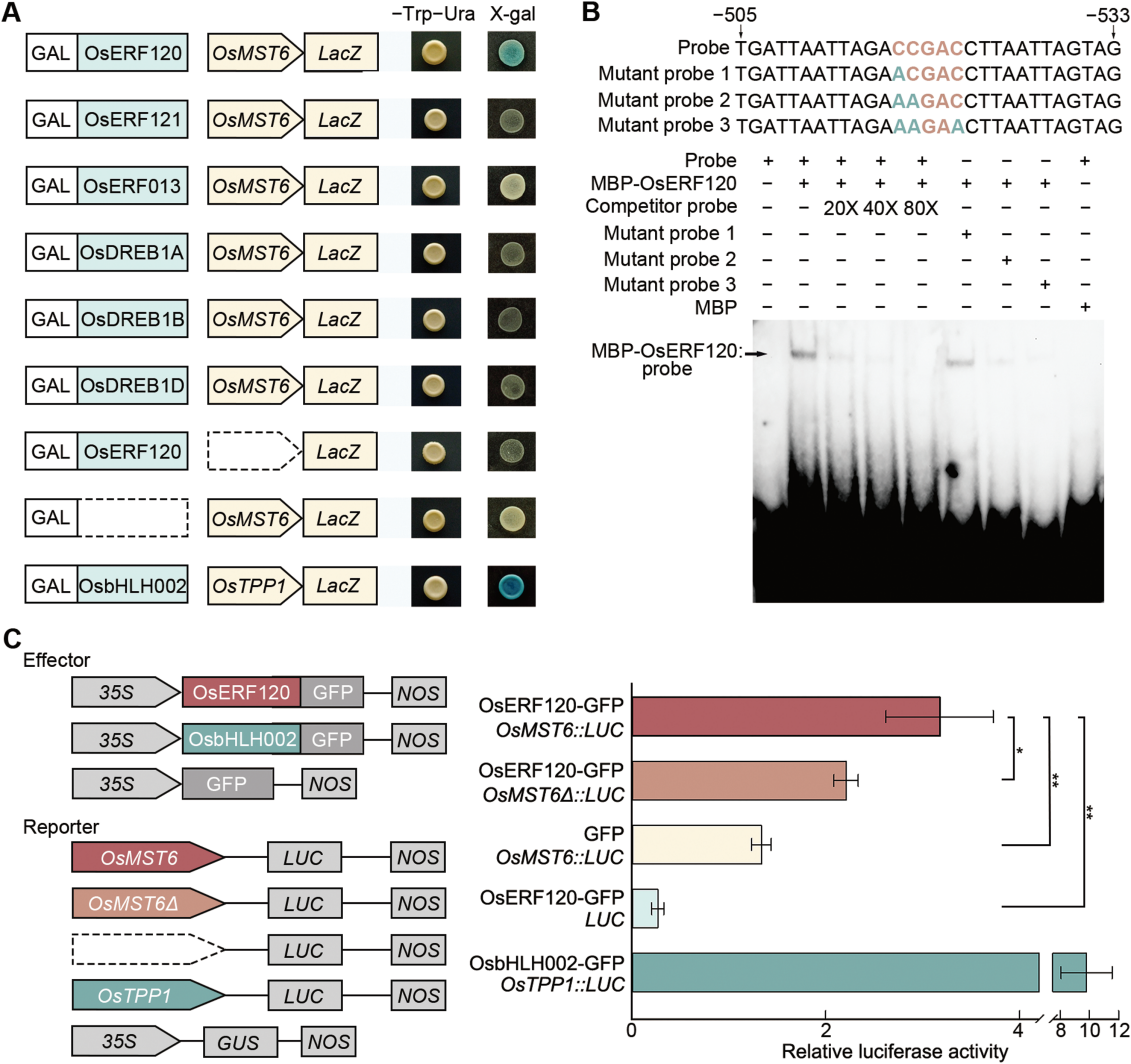
OsERF120 binds to the promoter of *OsMST6* and activates its transcription. (A) Yeast one-hybrid assays examining the abilities of OsERF120, OsERF121, OsERF013, OsDREB1A, OsDREB1B, and OsDREB1D to bind to the *OsMST6* promoter. OsbHLH002 and the *OsTPP1* promoter were used as the positive control. (B) Electrophoretic mobility shift assays (EMSA) demonstrating the binding of OsERF120 to the ‘CCGAC’ element in the promoter of *OsMST6*. The competitor probes were added at 20-, 40-, and 80-fold concentration of the labeled probes. The blue nucleotides represented the mutated nucleotides. (C) OsERF120 activated *OsMST6* transcription in rice protoplasts. Schematic diagrams for the effector and reporter are shown. *OsMST6Δ* indicates deletion of the ‘CCGAC’ element in the *OsMST6* promoter. OsbHLH002 and the *OsTPP1* promoter were used as the positive control. Data are means ±SD (*n=*3). Significant differences were determined by Student’s *t*-test, *P<0.05, **P<0.01.

To identify the key regulator of *OsMST6* expression, candidate transcription factors were predicted using the website PlantPAN v3.0 (Chow *et al.*, 2019). OsERF120, OsERF121, OsERF013, and DREBs, such as OsDREB1A, OsDREB1B, and OsDREB1D, were predicted to bind the *OsMST6* promoter. In yeast one-hybrid assays, the strain containing the OsERF120 and *OsMST6::LacZ* constructs was the first to turn blue (Fig. 4A). The strains co-expressing OsERF121, OsDREB1A, OsDREB1B, and OsDREB1D with *OsMST6::LacZ* also eventually turned blue, but needed more time than the strain containing OsERF120. Additionally, an electrophoretic mobility shift assay (EMSA) was employed to confirm the binding activity of OsERF120 to the *OsMST6* promoter. The fragment from −533 to −505 in the *OsMST6* promoter, containing the Dehydration Response Element (DRE)/C-repeat (CRT) element ‘CCGAC’, was used as the probe. The EMSA results showed a clear reduction in the migration rate of the OsERF120–probe complex relative to the control. As the unlabeled competitor probes gradually increased, the signal intensity of the OsERF120–probe complex gradually decreased. Mutating cytosines in the ‘CCGAC’ to adenine also resulted in reduced OsERF120–probe signal intensity (Fig. 4B). These results suggest that OsERF120 binds to the DRE/CRT element region of the *OsMST6* promoter.

To investigate the effect of OsERF120 transcriptional activity on the *OsMST6* gene, the luciferase (LUC) reporter gene was used to test the transcriptional activation activity. When OsERF120 and *OsMST6::LUC* were co-transfected into rice protoplasts, *OsMST6* transcription was activated. In contrast, the transcriptional activity of the mutated *OsMST6Δ*, which lacked the ‘CCGAC’ element, was significantly decreased compared with the complete *OsMST6* promoter (Fig. 4C). These results suggest that OsERF120 specifically binds to the DRE/CRT element of the *OsMST6* promoter and activates its transcription.

### OsERF120 positively regulates chilling stress responses through activation of *OsMST6*

**Fig. 5.**
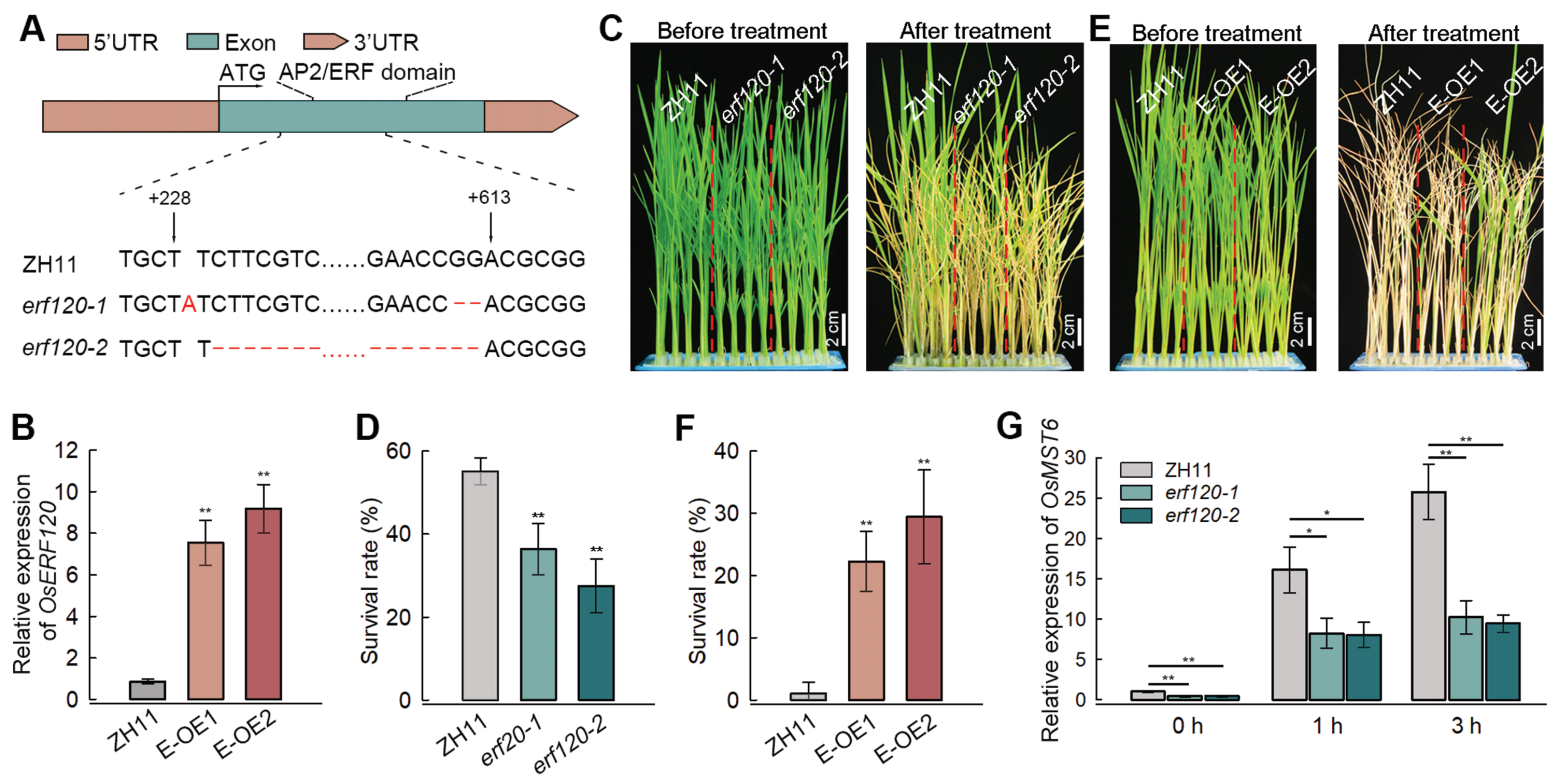
Chilling tolerance of *OsERF120* transgenic seedlings. (A) The gene structure and mutation sites in the *OsERF120* mutants (*erf120-1* and *erf120-2*). (B) Relative expression levels of *OsERF120* in the *OsERF120* overexpression lines (E-OE1, E-OE2) and the WT ZH11. The expression level of *OsERF120* in ZH11 was set as ‘1’. Data are means ±SD (*n*=3). (C) Phenotypes of ZH11, *erf120-1,* and *erf120-2* lines after chilling treatment. Scale bars, 2 cm. Fourteen-day-old seedlings were incubated at 4 °C for 90 h, then transferred to the greenhouse for recovery. (D) Survival rate of seedlings after 90 h of chilling treatment. Data are means ±SD (*n>*25 for each replicate). (E) Phenotypes of ZH11, E-OE1, and E-OE2 lines after chilling treatment. Scale bars, 2 cm. Fourteen-day-old seedlings were treated at 4 °C for 108 h, then transferred to the greenhouse for recovery. (F) The survival rate of seedlings after 108 h of chilling treatment. Data are means ±SD (*n>*25 for each replicate). (G) Relative expression levels of *OsMST6* in *erf120-1*, *erf120-2*, and ZH11 after chilling treatment for 0, 1, and 3 h. The expression level of *OsMST6* in ZH11 at 0 h was set as ‘1’. Data are means ±SD (*n*=3). Significant differences were determined by Student’s *t*-test, *P<0.05, **P<0.01.

OsERF120 was predicted to belong to the AP2/ERF superfamily, which is known to regulate diverse stress responses. Phylogenetic analysis of ERFs and DREBs showed that OsERF120 belonged to the same family as other ERFs, and was distinguished from DREBs (Supplementary Fig. S4A). Subcellular localization analysis indicated OsERF120 localized to the nucleus and cytoplasm (Supplementary Fig. S4B). To understand the genetic function of *OsERF120*, two knockout mutant lines, *erf120-1* and *erf120-2*, were generated using the CRISPR/CAS9 system in the ZH11 background. DNA sequencing results showed that *erf120-1* carried a single-base insertion behind the 228th nucleotide and two-base deletion in the AP2/ERF domain, resulting in frame-shift mutations, and *erf120-2* carried a deletion of 383 nucleotides, leading to premature termination (Fig. 5A; Supplementary Fig. S5A, B). At the same time, two overexpression lines of *OsERF120* (E-OE1 and E-OE2) were obtained. qRT-PCR analysis showed that the relative expression levels of *OsERF120* increased 7.5- and 9.1-fold, respectively, in the E-OE1 and E-OE2 lines, compared with ZH11 (Fig. 5B). Incubating rice seedlings at 4 °C for 90 h resulted in significantly reduced survival rates of *erf120-1* (36.4%) and *erf120-2* (27.5%), relative to ZH11 (55.1%) (Fig. 5C, D). Conversely, the survival rates of E-OE1 and E-OE2 were greater than 20%, while that of the ZH11 was reduced to less than 10% after 108 h chilling treatment (Fig. 5E, F). Since OsERF120 could directly bind to the *OsMST6* promoter (Fig. 4), the transcript levels of *OsMST6* were quantified. It was found that *OsMST6* transcription was induced by chilling stress in ZH11 and the *erf120* mutant. However, the expression levels of *OsMST6* were significantly reduced in both *erf120-1* and *erf120-2* lines after 0, 1, and 3 h of chilling treatment (Fig. 5G). In the overexpression lines, E-OE1 and E-OE2, driven by the constitutive CsVMV promoter, *OsMST6* expression was significantly up-regulated (Supplementary Fig. S5). These results suggest that *OsERF120* positively regulates chilling tolerance of rice seedlings by activating the transcription of *OsMST6*.

### OsMST6 affects sugar metabolism and hormone signaling

**Fig. 6.**
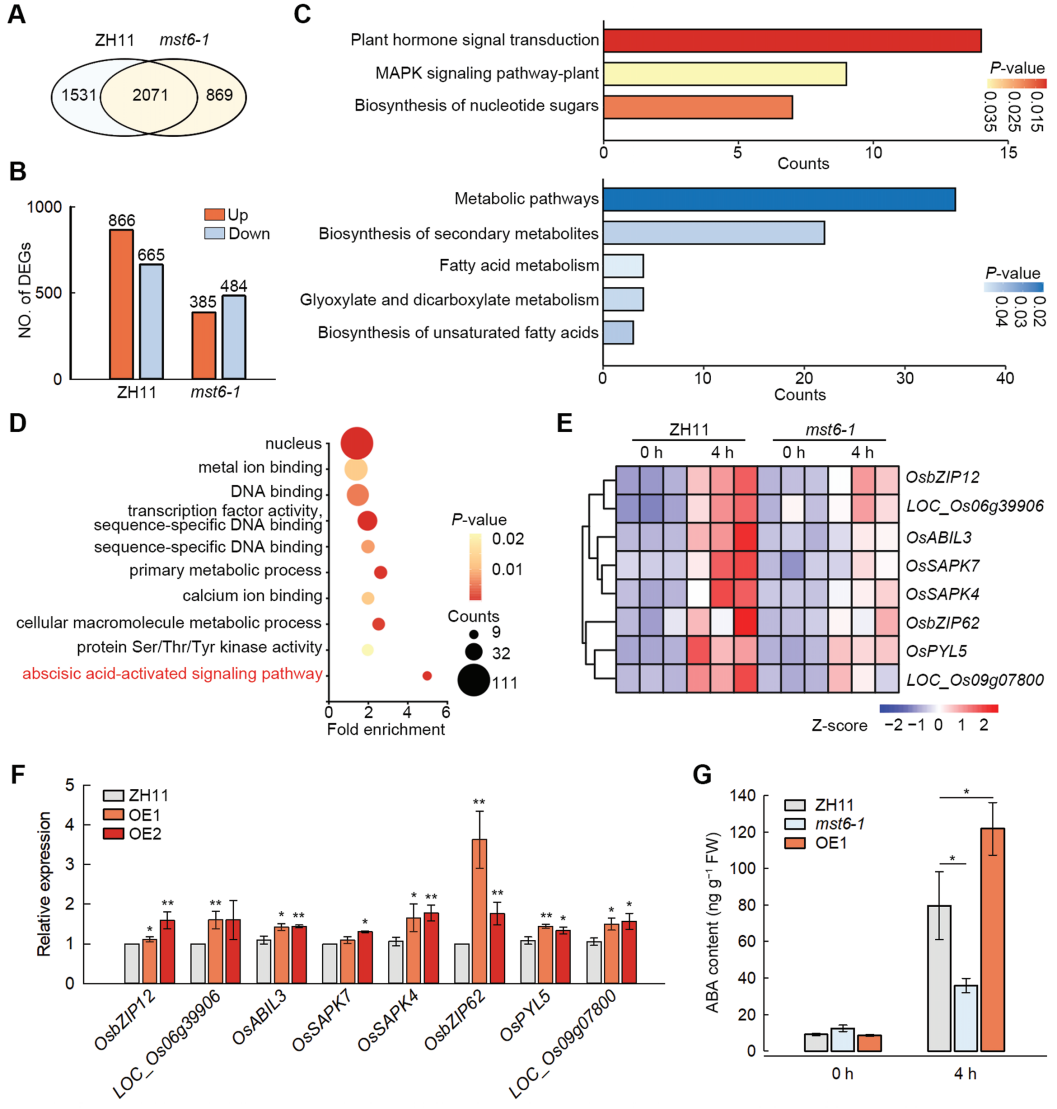
RNA-seq analysis of ZH11 and *mst6-1* seedlings. (A) Venn diagram showing the differentially expressed genes (DEGs) in ZH11 and *mst6-1*. |Fold change|≥2, *P*_adj_<0.05. (B) Number of unique DEGs in ZH11 and *mst6-1* after the 4 h chilling treatment. (C) Significantly enriched Kyoto Encyclopedia of Genes and Genomes (KEGG) terms of up-regulated DEGs in ZH11 and down-regulated DEGs in *mst6-1*. (D) The top 10 enriched gene ontology (GO) terms of up-regulated unique DEGs in ZH11. (E) Heatmap showing the transcriptional abundance of eight genes involved in the abscisic acid (ABA)-activated signaling pathway. (F) Relative expression levels of genes in the ABA-activated signaling pathway in OE1, OE2, and ZH11 lines after the 4 h chilling treatment. The expression level of ZH11 was set as ‘1’. Data are means ±SD (*n*=3). (G) The ABA content in whole rice seedlings after 0 h and 4 h of chilling treatment. FW, fresh weight. Data are means ±SD (*n=*3). Significant differences were determined by Student’s *t*-test, *P<0.05, **P<0.01.

To determine the effect of *OsMST6* mutation on transcriptomic changes during chilling stress, 14-day-old *mst6-1* and ZH11 rice seedlings exposed to chilling treatment were used for RNA-seq analysis. Compared with the control (0 h), 1531 unique differentially expressed genes (DEGs) were detected in the WT ZH11 seedlings after a 4 h treatment, while 869 unique DEGs were detected in the *mst6-1* seedlings (Fig. 6A). Among these unique DEGs, 866 were up-regulated and 665 were down-regulated in ZH11, while 385 genes were up-regulated and 484 genes were down-regulated in *mst6-1* (Fig. 6B). Among the 866 up-regulated unique DEGs in ZH11, the plant hormone signal transduction and mitogen-activated protein kinase (MAPK) signaling pathways were significantly enriched according to Kyoto Encyclopedia of Genes and Genomes (KEGG) analysis. Meanwhile, in the 484 down-regulated unique DEGs in *mst6-1*, several pathways involved in sugar metabolism showed significant enrichment, such as the fatty acid metabolism and biosynthesis of unsaturated fatty acids pathways (Fig. 6C). These results suggest that OsMST6 regulates rice chilling responses through plant hormone and sugar metabolism.

Among the up-regulated DEGs in ZH11, the most enriched pathway was the ABA-activated signaling pathway (Fig. 6D). Out of the 78 genes in the rice ABA-activated signaling pathway (GO:0009738), eight (10.3%) were up-regulated in ZH11 (Supplementary Fig. S6A; see also Dataset 3 at the Dryad Digital Repository, https://doi.org/10.5061/dryad.280gb5mw1). The heatmap indicated that the transcriptional abundance of these eight genes in *mst6-1* was lower than ZH11 after a 4 h chilling treatment (Fig. 6E). Furthermore, qRT-PCR results demonstrated that the majority of the eight genes were up-regulated in the OE1 and OE2 lines compared with ZH11 (Fig. 6F). Additionally, we noticed that among the up-regulated unique DEGs of ZH11, there were nine Protein Phosphatase 2C (PP2C) genes, accounting for 11.5% of all rice PP2C genes (Supplementary Fig. S6B). These results imply that OsMST6 positively regulates the ABA-activated pathway.

Quantification of ABA in whole rice seedlings showed that there was no significant difference in ABA content between the *mst6-1*, OE1, and ZH11 lines before chilling treatment. However, after 4 h of chilling treatment, the ABA content in *mst6-1* was reduced relative to the ZH11, and was increased in OE1 (Fig. 6G). Additionally, the application of exogenous ABA enhanced the chilling tolerance of the *mst6-1* and *mst6-2* mutants (Supplementary Fig. S7). These data imply that when chilling stress occurs, glucose transported into the cell by OsMST6 may serve as a precursor for metabolites and affect ABA signaling to enhance rice chilling tolerance.

## Discussion

STPs are conserved, 12-transmembrane domain, H^+^–sugar symporters, located on the plasma membrane (Deng *et al.*, 2019). However, STPs exhibit different expression patterns in different tissues and in response to different types of stresses, indicating potential functional differences (Kong *et al.*, 2019). The expression of *OsMST6* can be induced by salt and sugar applications, and OsMST6 exhibits glucose transporter activity when heterologously expressed in yeast (Wang *et al.*, 2008). Our data suggest that the expression of six STPs changed in response to the 4 h chilling treatment in rice seedlings, with *OsMST6* exhibiting the most significant increase in expression (Fig. 1B, C). Furthermore, in response to chilling stress, OsMST6 could transport glucose into rice cells, inducing *OsHXK2* and *OsNAC23* expression, and inhibiting the expression of *OsHXK7* (Fig. 3). Moreover, *mst6* mutants exhibited chilling sensitivity, whereas the overexpression lines showed increased chilling tolerance (Fig. 2). These results suggest that OsMST6 is a monosaccharide transporter that plays a crucial role in chilling stress responses in rice plants.

Hormone signaling networks can be activated by chilling stress, triggering the expression of chilling-regulated genes and coordinating developmental processes to enhance rice chilling tolerance (Eremina *et al.*, 2016). ABA plays a crucial role in plant growth, development, and response to abiotic stresses (Chen *et al.*, 2020). Chilling stress is also known to induce the accumulation of sugars (Chardon *et al.*, 2013; Le Hir *et al.*, 2015). Many sugar signaling-related genes have been reported to be involved in the ABA signaling pathway (Sakr *et al.*, 2018). In Arabidopsis, the ABA-insensitive (*abi4*) mutant is allelic to glucose insensitive 1 (*gin6*) (Matsoukas *et al.*, 2013). Mutants of *AtAIP1*, a critical gene in the ABA signaling pathway, exhibited reduced sensitivity to glucose treatment (Lim *et al.*, 2012). Our data suggests that in ZH11, there is a significant enrichment of the ABA-activated pathway (Fig. 6). Multiple ABA response-related genes, such as PP2Cs, which serve as the main ABA signaling node, are significantly up-regulated in ZH11, compared with *mst6* (Fig. 6E, Supplementary Fig. S6A, B). After chilling treatment, the ABA content in *mst6-1* was reduced relative to ZH11, while that in OE1 was increased (Fig. 6G). Moreover, exogenous ABA application rescues the cold tolerance of *mst6* (Supplementary Fig. S7). These data suggest that OsMST6 may enhance rice chilling tolerance through the ABA signaling pathway. Glucose in plants serves multiple functions, acting as an osmoprotectant, an energy source, a precursor of various metabolites, and a signaling molecule (Saddhe *et al.*, 2021). Our data indicate that in *mst6* mutants, as glucose transport into cells decreases, the expression of *OsHXK2* and *OsHXK7* is also affected (Fig. 3). At the same time, many sugar metabolism-related pathways, such as the fatty acid metabolism pathway, show significant decreases in expression in *mst6-1* (Fig. 6C). These results suggest that OsMST6 may play an important role in coordinating sugar and ABA signaling pathways in response to chilling stress in rice seedlings.

OsDREBs are key transcription factors involved in rice chilling stress responses (Novillo *et al.*, 2004; Ito *et al.*, 2006; Feng *et al.*, 2020). OsERFs and OsDREBs belong to the AP2/ERF superfamily, but functional studies on OsERFs have mainly focused on drought and submergence tolerance (Fukao *et al.*, 2006; Lee *et al.*, 2016; Jin *et al.*, 2018). Our data show that mutants of *OsERF120* are sensitive to chilling stress, while overexpression lines are more tolerant relative to the ZH11 (Fig. 5). DREBs and ERFs can both bind to the DRE/CRT sequence, but differences in flanking sequences can affect the binding preference (Feng *et al.*, 2019; Xie *et al.*, 2019). In *Vitis riparia*, the activation activity of VrCBF1 to the reporter genes was reduced, due to changes in the sequence flanking the DRE/CRT element (Nassuth *et al.*, 2014). In *Hordeum vulgare*, HvCBF1 has a binding preference for the ‘TTGCCGACAT’ element over the ‘ATGCCGACGT’ element (Xue, 2002). Our data showed that compared with OsDREB1A, OsDREB1B, and OsDREB1D, OsERF120 exhibited higher binding efficiency to the promoter of *OsMST6* (Fig. 4A). The preference of OsERF120 binding to the *OsMST6* promoter suggests that OsERF120 may be different from DREBs, and may represent a novel chilling tolerance regulatory pathway.

In summary, our data indicate that the OsERF120–OsMST6 pathway may represent a novel downstream pathway in the chilling tolerance regulatory network in rice seedlings. When rice seedlings are exposed to chilling stress, *OsMST6* expression is activated by OsERF120, resulting in increased glucose transport into cells, and coordinating sugar and ABA signaling pathways. These findings expand our understanding of the role of sugars in the chilling stress response and provide new insights into the downstream network of chilling tolerance mechanisms in rice.

## Supplementary data

The following supplementary data are available at *JXB* online.

Fig. S1. The alignment of protein sequences of OsMST6 in ZH11 and *mst6* mutants.

Fig. S2. The phenotype of *OsMST6* transgenic lines at the booting stage.

Fig. S3. The changes in relative fluorescence intensity under different concentrations of 2-NBDG.

Fig. S4. Phylogenetic analysis and subcellular localization of OsERF120.

Fig. S5. The alignment of protein sequences of OsERF120 in ZH11 and *erf120* mutants, and the expression of *OsMST6* in overexpression lines.

Fig. S6. ABA signal genes overlapped with DEGs in ZH11.

Fig. S7. The chilling phenotype of *mst6* after ABA treatment.

Table S1. List of primer sequences and accession numbers of genes used in this study.

erae123_suppl_Supplementary_Tables_S1_Figures_S1-S7

## Data Availability

The RNA-seq data underlying this article are available in the Genome Sequence Archive in National Genomics Data Center at https://ngdc.cncb.ac.cn/gsa, and can be accessed with accession number CRA011327. The datasets associated with this paper are openly available at the Dryad Digital Repository (Luo *et al*., 2024), https://doi.org/10.5061/dryad.280gb5mw1.

## Abbreviations

2-NBDG: 2-[*N*-(7-nitrobenz-2-oxa-1,3-diazol-4-yl) amino]-2-deoxy-d-glucose
AP2/ERF: APETALA2/ethylene-responsive element binding factor
DEG: differentially expressed gene
DRE/CRT: dehydration response element/C-repeat
HXK: hexokinase
LUC: luciferase
MST: monosaccharide transporter
STP: sugar transporter protein
ZH11: Zhonghua11.
