# Supplementary material for: Monosaccharide transporter OsMST6 is activated by transcription factor OsERF120 to enhance chilling tolerance in rice seedlings
Source: J Exp Bot. 2024 Mar 15;75(13):4038–51. doi: 10.1093/jxb/erae123 (PMC12351161; doi:10.1093/jxb/erae123)
Supplement: erae123_suppl_Supplementary_Tables_S1_Figures_S1-S7 [file erae123_suppl_supplementary_tables_s1_figures_s1-s7.pdf]

Supplementary data

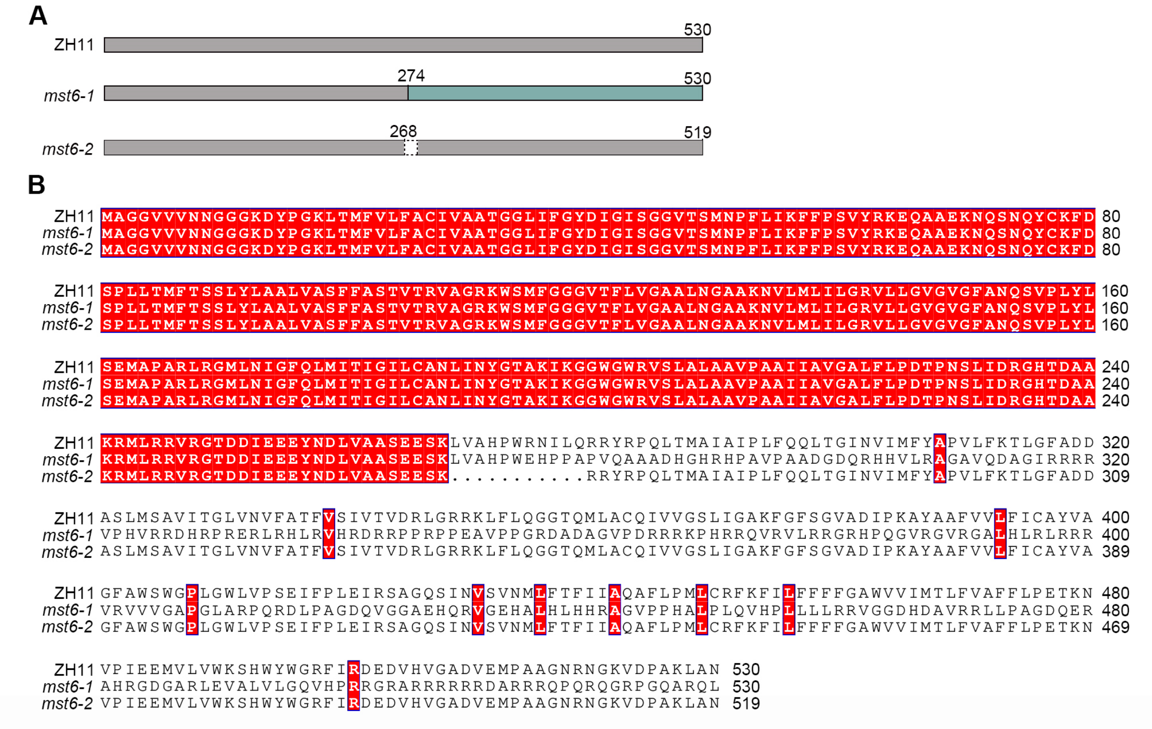

Fig. S1. The alignment of protein sequences of OsMST6 in ZH11 and *mst6* mutants

(A) The schematic representation of amino acid sequence in OsMST6 among the ZH11, *mst6-1*, and *mst6-2* lines. The different amino acid sequences were highlighted in green.

(B) The OsMST6 protein sequences of *mst6-1* and *mst6-2* mutants compared to the WT ZH11. The same amino acids are highlighted in red.

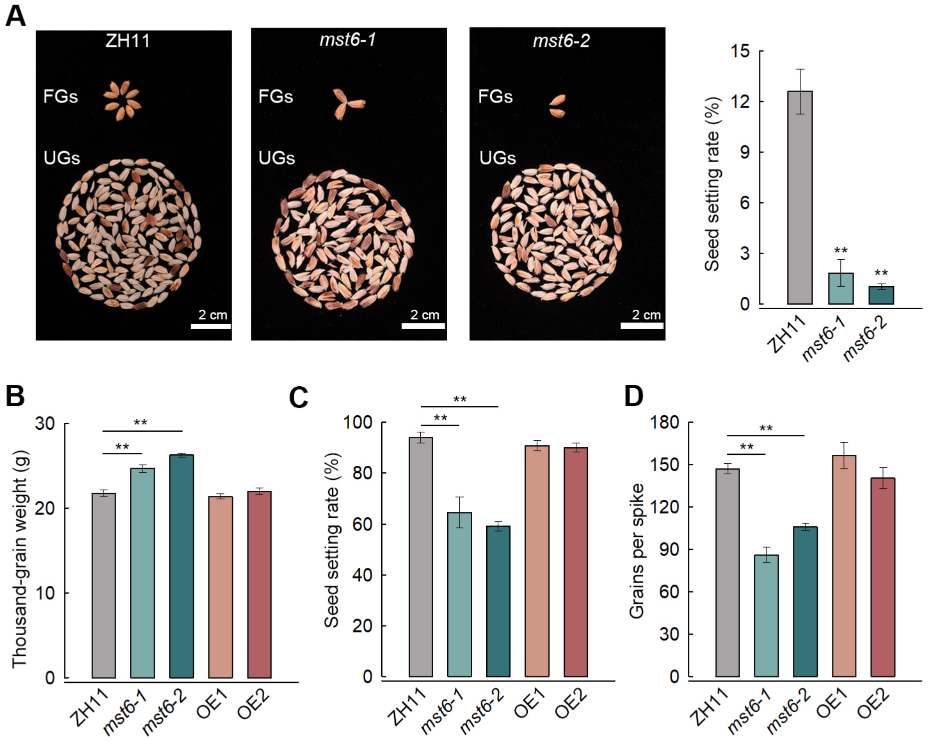

Fig. S2. The phenotype of *OsMST6* transgenic lines at the booting stage

(A) The evaluation of the seed setting rates in ZH11, *mst6-1*, and *mst6-2* lines after chilling treatment at the booting stage. Bar = 2 cm. FGs, filled grains; UGs, unfilled grains. Data are means ± SD (n = 3).

(B–D) The evaluation of agronomic traits of the *mst6-1*、*mst6-1*、OE1、OE2 and ZH11 lines under normal field conditions, including the thousand-grain weight (B), seed setting rate (C), and grains per spike (D).Significance: Student's t-test, $*P < 0.05$, $**P < 0.01$.

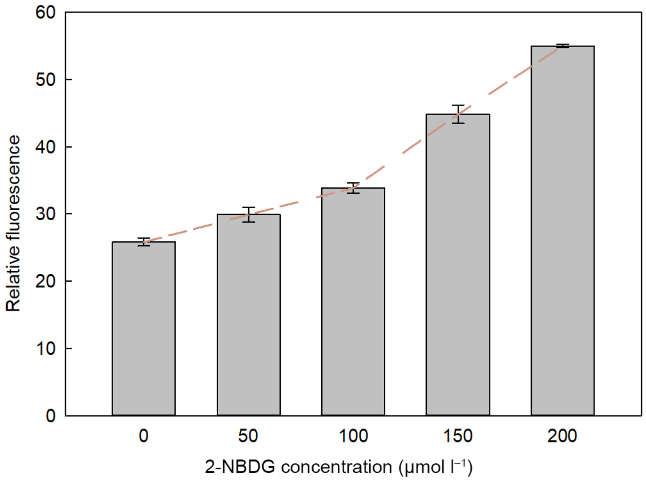

Fig. S3. The changes in relative fluorescence intensity under different concentrations of 2-NBDG

Rice protoplasts from ZH11 were incubated at concentrations of 0, 50, 100, 150, and 200 μmol l^{-1} for 1 h, and changes in intracellular fluorescence intensity were detected. The red dashed line represents the trend of relative fluorescence intensity.

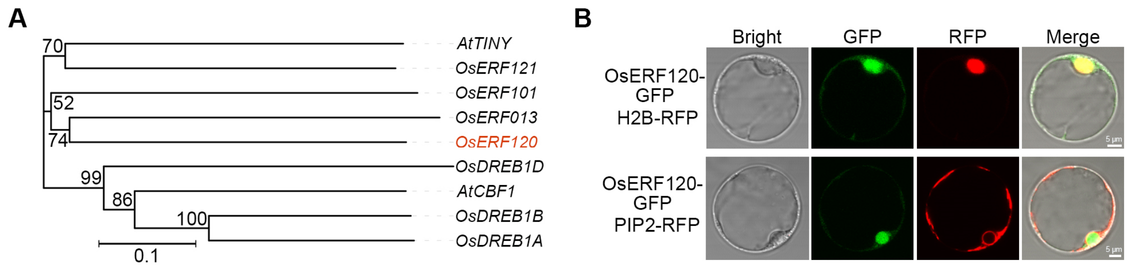

Fig. S4. Phylogenetic analysis and subcellular localization of *OsERF120*

(A) Phylogenetic analysis of *OsERF120*, other ERF family members (*OsERF013*, *OsERF101*, *OsERF121*, *AtTINY*), and DREBs (*OsDREB1A*, *OsDREB1B*, *OsDREB1D*, *AtCBF1*).

(B) Subcellular localization of OsERF120 in rice protoplasts. H2B was a nucleus marker and PIP2 was a membrane marker. Bar = 5 μm.

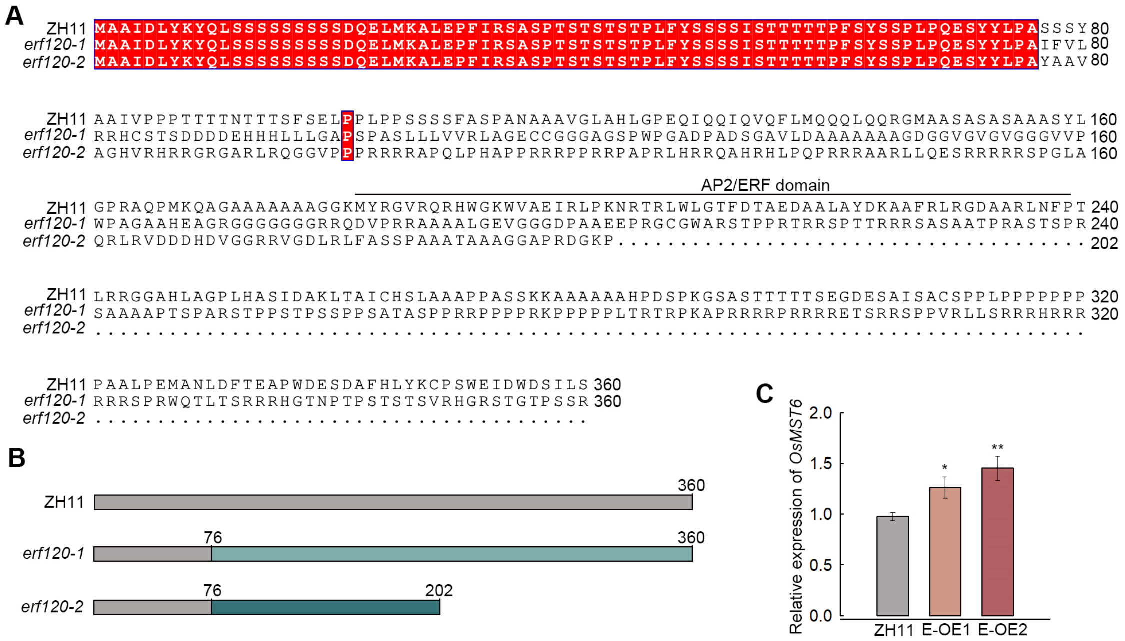

Fig. S5. The alignment of protein sequences of OsERF120 in ZH11 and *erf120* mutants, and the expression of *OsMST6* in overexpression lines

(A) The OsERF120 protein sequences of *erf120-1* and *erf120-2* mutants compared to the WT ZH11. The same amino acids are highlighted in red. The AP2/ERF domain of OsERF120 in ZH11 has been delineated by the black line.

(B) The schematic representation of amino acid sequence in OsERF120 among the ZH11, *erf120-1*, and *erf120-2* lines. The different amino acid sequences were highlighted in green.

(C) Relative expression levels of *OsMST6* in the E-OE1, E-OE2 and ZH11 lines. The expression level of *OsMST6* in ZH11 was set as “1”. Data are means ± SD (n = 3). Significance: Student’s t-test, $*P < 0.05$, $**P < 0.01$.

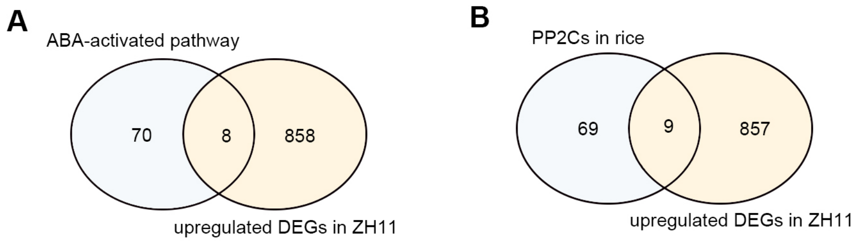

Fig. S6. ABA signal genes overlapped with DEGs in ZH11.

(A) Venn diagram showing the genes of ABA-activated signaling pathway overlapped with 866 unique up-regulated DEGs in ZH11 after 4-hour chilling treatment.

(B) Venn diagram showing that all PP2Cs in rice overlapped with 866 unique up-regulated DEGs in ZH11 after 4-hour chilling treatment.

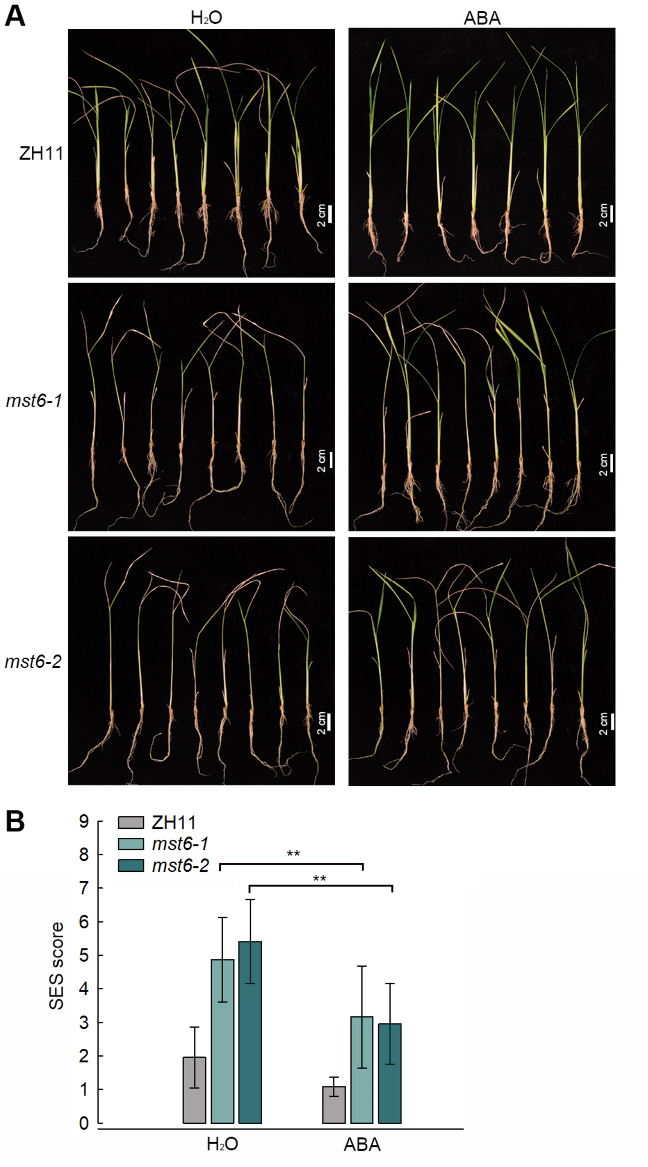

Fig. S7. The chilling phenotype of *mst6* after ABA treatment

(A) The phenotype of the *mst6-1*, *mst6-2*, and ZH11 lines after the 72-hour chilling treatment following exogenous ABA application and water control. Bar = 2 cm.

(B) The IRRI SES score of individual plants after 72-hour chilling treatment. Data are means ± SD (n = 25). Significance: Student's t-test, $^{**}P < 0.01$.

Table S1. List of primer sequences and accession numbers of genes used in this study.

Primers for CRISPR/CAS9 genome editing vector	
cOsMST6-F	TGTGTGGCGGAACATCCTCCAG
cOsMST6-R	AAACTGGAGGATGTTCCGCCAC
cOsERF120-F	GCAGGTCTCATGTGGCTGCCGAAGAACCGGACGGTTTTAGAGCTAGAAATAGCAAGTT
cOsERF120-R	GCAGGTCTCTAAAACGCTTCTTCGTCCTACGCCGCTGCCACGGATCATCTGCA
Primers for overexpression lines constructs	
OsMST6-F-XbaI	GCTCTAGAATGGCCGGCGGCGTGGTGGT
OsMST6-R-KpnI	GGGGTACCGTTGGCGAGCTTGGCCGGGT
OsERF120-F-recom	GTAGATCTTAATTAATCTAGAGAATTCATGGCGGCCATAGATTTGTA
OsERF120-R-recom	CCATGGATCCCGGGCCCGCGGTACCCGAGAGGATGGAGTCCCAGT
Primer for subcellular localization	
OsMST6-F-recom-221	ACTCTAGAGGATCTCGAGATGGCCGGCGGCGTGGTGGT
OsMST6-R-recom-221	TCCTTTACCCATGGTACCGTTGGCGAGCTTGGCCGGGT
Primers for quantitative RT-PCR	
qOsMST6-F	ACAACGGAGGGGGGAAGGACTAC
qOsMST6-F	TTGATCAGGAACGGGTTCATCGAC
qOsERF120-F	AAACCTTGACTTCACGGAGGC
qOsERF120-R	CGAGAGGATGGAGTCCCAGT
qUBI-F	AGGGTTCACAAGTCTGCCTATTT
qUBI-R	TCTTCCATGCTGCTCTACCACA
qActin-F	CAGCCACACTGTCCCCATCTA
qActin-R	AGCAAGGTCGAGACGAAGGA
qOsHXK2-F	GATGCACTGCGACAGATCAC
qOsHXK2-R	CAGACATCCACCACAAGCCT
qOsHXK7-F	ACCTTCTCTTTCCCTGTGCG
qOsHXK7-R	CATGGCCATCTGCAATTCGG
qOsNAC23-F	ATGAACTCTCCAAGAGCGGC
qOsNAC23-R	ACAGCACCATCTCTTCCGTG
q6g39906-F	AGGAAATAGAGCCCTCTCCAGT
q6g39906-R	GTGGAAGGCTTGTCACTTGAAC
qABIL3-F	CACCATTGTCTGATGAGGGTGA
qABIL3-R	TGTTATCCTCGCTGCCTTTCTT
qSAPK4-F	AGCCTACCCATTTGAAGACCAG
qSAPK4-R	AAATGCGGGCAATAAGCTGTTT
qPYL5-F	ATCCATCGTAACTGTCCATCCG
qPYL5-R	GGCGAGAGATGTTAAGTTGCAC
qPP2C41-F	CAGACCAAGCAATCCTGTCA

qPP2C41-R	CAAAGCCACCCCTATTTTCA
Primers for yeast one hybrid	
OsERF120-F-recom-pGAD42	ATTATGCCTCTCCCGAATTCATGGCGGCCATAGATTTGTA
OsERF120-R-recom-pGAD42	GAAGTCCAAAGCTTCTCGAGCGAGAGGATGGAGTCCCAGT
OsERF121-F-recom-pGAD42	ATTATGCCTCTCCCGAATTCATGCGGAAGTCGAAGCAGCC
OsERF121-R-recom-pGAD42	GAAGTCCAAAGCTTCTCGAGGAGGCGGAGCAGCGTGTCGT
OsERF013-F-recom-pGAD42	ATTATGCCTCTCCCGAATTCATGGCGTACATCAGTACATC
OsERF013-R-recom-pGAD42	GAAGTCCAAAGCTTCTCGAGGACATCGAAGCTCCAAAGCT
OsDREB1A-F-recom-pGAD42	GCCAGATTATGCCTCTCCCGAATTCATGTGCGGGATCAAGCAGGA
OsDREB1A-R-recom-pGAD42	GCGAAGAAGTCCAAAGCTTCTCGAGCTAGTAGCTCCAGAGTGGGAC
OsDREB1B-F-recom-pGAD42	GCCAGATTATGCCTCTCCCGAATTCATGGAGGTGGAGGAGGCGGC
OsDREB1B-R-recom-pGAD42	GCGAAGAAGTCCAAAGCTTCTCGAGTTAGTAGCTCCAGAGCGGCAT
OsDREB1D-F-recom-pGAD42	GCCAGATTATGCCTCTCCCGAATTCATGGAGAAGAACACCGCCGC
OsDREB1D-R-recom-pGAD42	GCGAAGAAGTCCAAAGCTTCTCGAGCTACCTCCTCCATTGAAAAAG
OsMST6-F-LacZ-HindIII	CCCAAGCTTGGGTACCTGAAAGGACTGTAAAT
OsMST6-R-LacZ-XhoI	CCGCTCGAGCGGCATAAAGAAAAGATCATGGA
Primers for EMSA	
OsERF120-F-recom-MAL	GGGAAGGATTTCAGAATTCGGATCCATGGCGGCCATAGATTTGTA
OsERF120-R-recom-MAL	GTGCCAAGCTTGCCTGCAGGTCGACTCACGAGAGGATGGAGTCCC
Probe-F	TGATTAATTAGACCGACCTTAATTAGTAG
Probe-R	CTACTAATTAAGGTCGGTCTAATTAATCA
Mutant probe1-F	TGATTAATTAGAACGACCTTAATTAGTAG
Mutant probe1-R	CTACTAATTAAGGTCGTTCTAATTAATCA
Mutant probe2-F	TGATTAATTAGAAAGACCTTAATTAGTAG
Mutant probe2-R	CTACTAATTAAGGTCTTTCTAATTAATCA
Mutant probe3-F	TGATTAATTAGAAAGAACTTAATTAGTAG
Mutant probe3-R	CTACTAATTAAGTTCTTTCTAATTAATCA

Primers for transcription activity assays	
OsMST6-F-recomLUC	CCTGCAGGTCGACTCTAGAGGATCCGTAGGAAGACGAACAATCCC
OsMST6-R-recomLUC	TGTTTTTGGCGTCTTCCATGGTACCAAAGAAAAGATCATGGACAA
OsERF120-F-recom-221	ACGGGGGACTCTAGAGGATCTCGAGATGGCGGCCATAGATTTGTA
OsERF120-R-recom-221	GTTCTTCTCCTTTACCCATGGTACCCGAGAGGATGGAGTCCCAGT
Gene name	ID
OsMST6	*LOC_Os07g37320*
OsERF120	*LOC_Os06g11860*
OsERF121	*LOC_Os06g47590*
OsERF013	*LOC_Os06g11940*
OsERF101	*LOC_Os04g32620*
OsDREB1A	*LOC_Os09g35030*
OsDREB1B	*LOC_Os09g35010*
OsDREB1D	*LOC_Os06g06970*
Ubiquitin	*LOC_Os06g46770*
Actin	*LOC_Os03g50885*
AtTINY	*At5g25810*
AtCBF1	*At4g25490*
